# Folding of Trp-cage Mini Protein Using Temperature and Biasing Potential Replica—Exchange Molecular Dynamics Simulations

**DOI:** 10.3390/ijms10031121

**Published:** 2009-03-12

**Authors:** Srinivasaraghavan Kannan, Martin Zacharias

**Affiliations:** School of Engineering and Science, Jacobs University Bremen, Campus Ring 1, D-28759 Bremen, Germany

**Keywords:** Conformational sampling, molecular dynamics simulation, protein folding, Replica Exchange

## Abstract

The folding process of the 20 residue Trp-cage mini-protein was investigated using standard temperature replica exchange molecular dynamics (T-RexMD) simulation and a biasing potential RexMD (BP-RexMD) method. In contrast to several conventional molecular dynamics simulations, both RexMD methods sampled conformations close to the native structure after 10–20 ns simulation time as the dominant conformational states. In contrast, to T-RexMD involving 16 replicas the BP-RexMD method achieved very similar sampling results with only five replicas. The result indicates that the BP-RexMD method is well suited to study folding processes of proteins at a significantly smaller computational cost, compared to T-RexMD. Both RexMD methods sampled not only similar final states but also agreed on the sampling of intermediate conformations during Trp-cage folding. The analysis of the sampled potential energy contributions indicated that Trp-cage folding is favored by both van der Waals and to a lesser degree electrostatic contributions. Folding does not introduce any significant sterical strain as reflected by similar energy distributions of bonded energy terms (bond length, bond angle and dihedral angle) of folded and unfolded Trp-cage structures.

## 1. Introduction

Realistic computer simulation of the structure formation process of proteins is a great challenge of molecular biophysics and structural biology. The prediction of protein structures and of the folding process at an atomic level of detail can help to understand the function of proteins and may allow the creation of new macromolecules with desired functions.

In recent years, molecular dynamics (MD) simulations have been widely used for studying folding processes of peptides and small proteins, including the characterization of folding pathways and intermediate states [1–5]. However, the currently possible time scales of ~100 ns still limit the applicability of MD simulation to small proteins and simulation times that are insufficient to study the folding process systematically. Proteins and peptides can adopt numerous locally stable conformations that are separated by large energy barriers. At room temperature a standard MD simulation may easily be trapped into a locally stable conformation. Conformational transitions between these stable states are rare during conventional MD (cMD) simulations [1–6]. A variety of methods like simulated annealing [7], potential scaling [8–15], locally enhanced sampling [16], or parallel tempering [17–19] have been proposed to overcome the conformational sampling problem during molecular simulations (reviewed in [5,6]). A standard method to enhance conformational sampling uses simulated annealing (SA-MD), that is, to start with simulations at high temperature to overcome barriers followed by gradual cooling (annealing) to reach low energy regimes [20]. Unfortunately, during SA-MD a given protein or peptide system may still be trapped in a local energy minimum and the decrease in temperature will further lower the escape probability to explore new alternative low energy regions. Except for excessively long simulations there is no guarantee that simulated annealing will find a global minimum [21].

Another method used to enhance conformational sampling in both Monte Carlo (MC) and MD simulations is the parallel tempering or replica exchange approach [17–19,22–30]. In RexMD simulations several copies (replicas) of the system are simulated independently and simultaneously using classical MD at different simulation temperatures. At preset intervals pairs of replicas (neighboring pairs) are exchanged with a specified transition probability. Typically, temperature is used as a parameter to be varied and exchanged among the replicas (T-RexMD). The random walk in temperature allows conformations trapped in locally stable states (at a low simulation temperature) to escape by exchanging with replicas at higher simulation temperature. An advantage of RexMD compared to SA-MD is that exchanges between low and high temperature regimes are possible throughout the whole simulation combining efficiently the barrier crossing properties at high temperature with the selectivity for low energy structures at low temperature. The RexMD method has been successfully applied in folding simulations of several peptides and small proteins [22–30]. A drawback of RexMD is, however, the large number of simulations that run in parallel in order to cover a desired temperature range. Since efficient exchanges between neighboring replicas require sufficient energy overlap between replicas the number of required replicas grows rapidly with the size of the simulation system (~with the square root of the number of particles, [31]). A large number of replicas in turn require increased simulation times in order to allow efficient “traveling” of replicas in temperature space.

As an alternative to the use of temperature as a replica coordinate, it is also possible to use the force field or Hamiltonian of the system as parameter that differs between replicas [31–36]. Recently, we have proposed a Hamiltonian replica exchange (H-RexMD) method termed biasing potential – Replica Exchange (BP-RexMD), that focuses on the protein backbone transitions. The method employs a biasing potential to reduce the energy barriers associated with peptide backbone dihedral transitions. The level of added biasing is gradually changed along the replicas such that frequent transitions are possible at high levels of biasing and the system can escape from getting trapped in local energy minima. Since exchanges between replicas depends only on the part of the system that involves the biasing potential the method requires fewer replicas for efficient sampling compared with standard temperature (T-)RexMD. Indeed, the application of BP-RexMD on small peptides showed improved sampling of conformational space with fewer replicas (only 5–7) as compared to standard temperature RexMD simulations [37].

In the current study the BP-RexMD has been applied to the folding of Trp-cage mini protein in implicit solvent. The study includes a direct comparison of sampled states with T-RexMD and an energetic analysis of driving forces for folding based on a continuum solvent model. The Trp-cage is a 20 residue mini-protein designed by Neidigh *et al.* [38] based on the C-terminal fragment of the 39-residue exendin-4 peptide. The structure of this protein was determined by NMR spectroscopy [38]. The Trp-cage protein contains different types of secondary structure and a characteristic well structured hydrophobic core where the indole side chain of a Trp residue is buried between the rings of two Pro residues. Its folding behavior has been investigated by various experimental methods. Qui *et al.* [39] suggested a two-state folding mechanism based on laser temperature jump spectroscopy whereas Ahmed *et al*. [40] using UV- resonance Raman spectroscopy measurements suggested a more complicated folding mechanism through an intermediate molten globule state. The study also provided evidence for α-helical structure even in the denatured state of the Trp-cage protein. Recently, Mok *et al*. [41] found extensive hydrophobic contacts, even in the unfolded state, employing photochemically induced dynamic nuclear polarization (CINDP)-NMR pulse-labeling experiments. It needs to be emphasized that the force field accuracy is an additional factor which limits the ability of current simulation approaches to reproduce protein folding events. Furthermore, the present simulation study employed an implicit (Generalized Born-type) solvation model. Nevertheless, such models have already been used successfully for simulating the folding of the Trp-cage protein during either conventional MD simulations [42–45] or T-RexMD [46]. However, an analysis of energetic contributions to the folding process has so far not been provided.

## 2. Materials and Methods

For all MD simulations the *Sander* module of the AMBER9 package [47] was used in combination with the parm03 force field [48] and an advanced Generalized Born implicit solvent model (GB-option=5 in *Sander*) according to Onufriev *et al*. [49]. An initial extended structure for the Trp-cage protein was generated using the xleap module of the Amber9 package [47]. The simulation system was subjected to energy minimization (1,000 steps) using the sander module. During MD simulation, the protein was initially harmonically restrained (25 kcal mol^−1^Ǻ^−2^) to the energy minimized start coordinates, and the system was heated up to 300 K in steps of 100 K followed by gradual removal of the positional restraints and a 0.5 ns unrestrained equilibration at 300 K. The resulting system was used as starting structure for both BP-Rex MD and T-Rex MD and all conventional MD simulations. A time step of 2 fs were used for the BP-RexMD simulations whereas for T-Rex MD simulation it was necessary to reduce the time step to 1 fs.

In standard temperature T-RexMD, copies or replicas of the system are simulated simultaneously at a set of temperatures (T_0_,T_1_,T_2_,….T_N_). Each of the copies evolves independently and after preset time intervals exchanges of pairs of neighboring replica are attempted according to a Metropolis criterion [18]:

$$\begin{array}{l} w(x_{i}\to x_{j})=1\;\;\;\;\;\text{for}\Delta \leq 0;\\ w(x_{i}\to x_{j})=\text{exp}(-\Delta )\;\;\text{for}\Delta >0\\ \text{where}\\ \Delta =(\beta_{i}-\beta_{j})\left[E(r_{j})-E(r_{i})\right] \end{array}$$

with β=1/RT (R: gas constant and T: temperature) and E(r) representing the potential energy of system for a given configuration. In the current work an exchange was attempted for every 2,000 steps (2 ps). It has been recognized that temperature (represented as Boltzmann factor β) and energy (or Hamiltonian of the system) are equivalent in the Metropolis criterion. The BP-RexMD method is a Hamiltonian RexMD method that employs a biasing potential for the Φ and Ψ peptide backbone dihedral angles [37]. The biasing potential is based on a potential of mean force (PMF) for each of the two dihedral angles calculated for a model peptide (alanine dipeptide) in explicit solvent [37]. Addition of the biasing potential during a simulation lowers the energy barriers for backbone dihedral transitions in a peptide or protein. In a BP-RexMD simulation different biasing potential levels are applied in each replica (one reference replica runs without any biasing potential) and replica exchanges between neighboring biasing levels were attempted every 1,000 MD steps (2ps) and accepted or rejected according to a Metropolis criterion [18]:

$$\begin{array}{l} w(x_{i}\to x_{j})=1\;\;\;\;\;\text{for}\Delta \leq 0;\\ w(x_{i}\to x_{j})=\text{exp}(-\Delta )\;\;\text{for}\Delta >0\\ where\\ \Delta =\beta \left[\left(E^{j}(r_{j})-E^{j}(r_{i})\right)-\left(E^{i}(r_{i})-E^{i}(r_{j})\right)\right] \end{array}$$

Here, the Metropolis criterion involves only a single β or temperature (in the present study 300K) and the energy difference between neighboring configurations using the force field for replica j (E^j^) minus the same difference using force field for replica i (E^i^). An advantage compared to temperature RexMD is the fact the energy differences are only affected by the force field term that changes upon going from one replica to another replica run.

For comparison, five conventional cMD simulations (with different initial velocities) were carried out starting from the same structure. Both the cMD simulations and the BP-RexMD simulations were carried at a temperature of 300 K. For the T-Rex MD simulation a temperature range of 300 K – 460 K was used (temperatures in Kelvin: 300, 303, 307, 312, 318, 325, 332, 339, 348, 360, 374, 390, 406, 422, 440, 460). The exchange acceptance rate for the T-RexMD was between 20–35%. For the BP–RexMD simulation five replicas with the same biasing potential and biasing levels as given in Table I of reference [37] were used. The complete BP-RexMD simulation required less than two weeks of computer time using eight nodes of a cluster computer for each replica. The acceptance probability for replica exchanges was in the range of 30–40%. Cluster analysis of sampled conformation was performed using the kclust program in the MMTSB-tools [50] base on root-mean-square deviation of Ca-atoms (Rmsd_Ca_) with a 2 Å cutoff. Structures were visualized using VMD [51].

## 3. Results and Discussion

### 3.1. Comparison of continuous and Replica Exchange MD simulations

Five continuous MD simulations at 300K were started from an extended Trp-cage protein structure generated using the Amber leap module and assigning different initial velocities. In addition to the simulations started from extended conformations, one 40 ns control simulation run starting from the folded structure (first entry of pdb1L2Y) was also performed. The deviation of the sampled structures for this simulation remained mostly to within ~2 Å (heavy atom Rmsd: Rmsd_heavy_) from the experimental structure (not shown).

**Figure 1.**
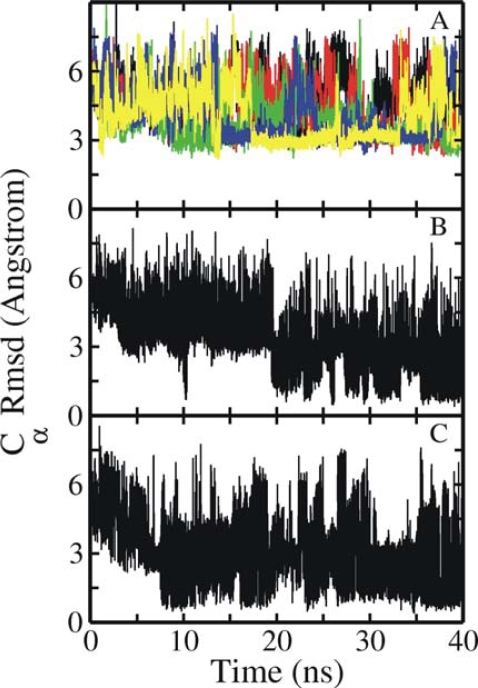
Root-mean-square deviation (Rmsd of Cα-atoms) of sampled Trp-Cage conformations from the native structure (1^st^ entry of pdb1L2Y) vs. simulation time. (A) Rmsd of five independent cMD simulations starting from an extended Trp-cage structure with different initial atomic velocities. (B) Rmsd of conformations sampled during the T-RexMD started from the same extended conformation (for the reference replica run). (C) same as in (B) but for the BP-RexMD (for the replica run with the original force field).

None of the five independent simulations starting from the unfolded conformation resulted in structures with a backbone Rmsd_Cα_ < 2.5 Å or Rmsd_heavy_ < 4 Å within the 40 ns simulation time (Figure 1a). Even an extension to 100 ns of two of the cMD simulations did not result in conformations in closer agreement with the experimental structure (not shown). In addition to cMD simulations a BP-RexMD simulation (five replicas including always one simulation that runs without a biasing potential) and a T-RexMD simulation (16 replicas) were carried out starting from the same initial unfolded conformation. The BP-RexMD method employs a specific biasing potential to promote peptide backbone transitions as a replica coordinate. The purpose of the biasing potential is to reduce the energy barriers associated with peptide backbone dihedral transitions [37]. Note, that the same biasing potential was used for all backbone dihedral angles in the Trp-cage protein (except Gly and the Pro Φ angle). The derivation of the biasing potential has been described in reference [37] and the same biasing levels were used as given in Table I of reference [37]. The T-RexMD simulations were performed using 16 replicas with temperatures ranging from 300 K to 460 K (see Methods). Exchanges were attempted for both RexMD methods at every 2 ps between neighboring replica and extended to up to 40 ns (20,000 attempted exchanges).

In contrast to the continuous MD simulations in both the replica exchange simulations the deviation of the sampled conformations from the native structure (in the reference replica) dropped to Rmsd_Cα_ < 1 Å within the first 10 ns of simulation time (Figures 1b,c). However in the T-Rex MD simulation conformations close the folded structure disappeared and started to accumulate again after ~20 ns time period (Figure 1b). Structures < 1.5 of Rmsd_Cα_ and ~2 Å Rmsd_heavy_ from the native structure were sampled as the dominant state in the reference replica of both the RexMD simulations during the final 10–15 ns time period. The near-native structures also showed very good agreement with NMR-derived proton distances in the folded state (not shown).

**Figure 2.**
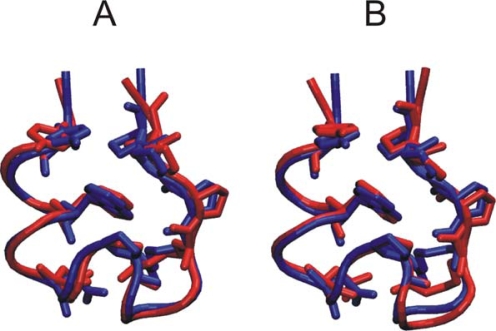
Superposition of the cluster centroids (structure closest to the average structure of the cluster) from the most populated cluster during the final part (last 10 ns) of the BP-RexMD (A) and T-Rex MD (B) simulations (red structures) on the native Trp-cage structure (first entry of pdb1L2Y; in blue). The protein backbone is in tube representation and residues Tyr_3_, Trp_6_, Asp_9_, Pro_12_, Arg_16_, Pro_17_, Pro_18_ and Pro_19_ are shown as stick model.

The centroid (conformer closest to the center of a cluster) representing the most populated state of the final phase of both BP-Rex MD and T-Rex MD simulation had an Rmsd_Cα_ of 0.9 and 1.1 Å, respectively, from the experimental structure (Figure 2a,b). However, structures with an Rmsd_Cα_ as close as 0.4 Å from the experimental structure were sampled during the final phase of the both simulations.

### 3.2. Comparison of conformational sampling during T-RexMD and BP-RexMD

Both the BP-RexMD as well as the T-RexMD simulations resulted in structures with similar Rmsd form experiment at the final stage of the simulations (Figure 1). Note, that the BP-RexMD method achieved this at a fraction of the computational cost of the T-RexMD method (five vs. 16 replicas). In addition, cluster analysis of the sampled structures indicated that near native structures of the Trp-cage protein form in both cases the most dominant cluster at the final part of the simulations. The population of the cluster representing near native Trp-cage structures reached 45% and 40% in the BP-Rex MD and T-RexMD simulations, respectively.

The mechanism of enhanced sampling of the two RexMD methods differs due to the use of temperature or scaling of specific force field terms as replica parameter. Although both approaches preserve detailed balance during exchanges between neighboring replicas and therefore should achieve a canonical sampling of all relevant states for sufficiently long simulations it is of significant interest if both methods also sample similar intermediate states during the relatively small (40 ns) simulation time.

**Figure 3.**
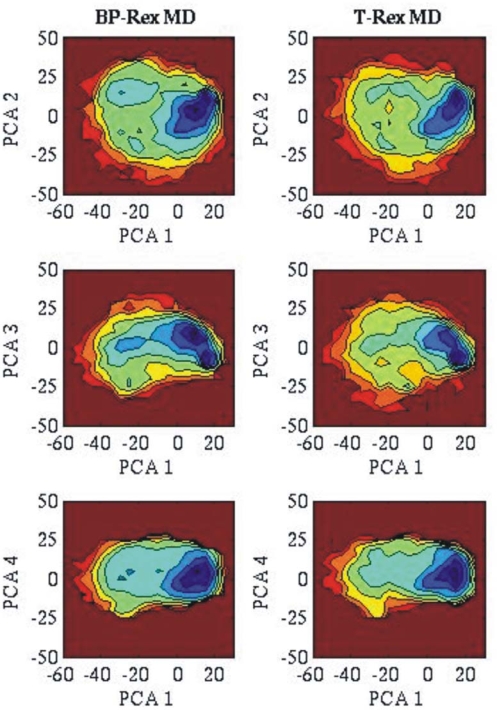
Projection of sampled states (in reference replicas) on 2-dimensional planes spanned by PC 1 (x-axis) and PCs 2–4 (y-axis). The PCs were obtained from the analysis of the BP-RexMD simulations (reference replica). The projected distribution is shown as the free energy contour plot (logarithm of density, blue color indicates favorable regions).

To further compare the sampling efficiency of BP-RexMD and T-RexMD simulations, principal component analysis was carried out for the positional co-variation of both types of simulation (for reference replica runs). The structures that were sampled during the BP-Rex MD simulation were used for calculating principal components (PCs). The complete trajectories from the reference replica of both the replica simulations were projected onto 2D planes spanned by combinations of soft PCs (with largest eigenvalues or largest contribution to conformational fluctuations). It should be mentioned that the soft PCs calculated from the BP-RexMD showed significant overlap with the corresponding PCs from the T-RexMD simulation. Projection of the three softest PCs from the T-RexMD onto the subspace spanned by the first five softest PCs from the BP-RexMD (or vice versa) indicated an overlap of > 80 %. The correlation graphs between different PCs were converted into 2D free energy contour maps (logarithm of the probability distributions in units of RT). As illustrated in Figure 3, the projected distribution of sampled conformers in 2D contour maps of most relevant principal components looks very similar for both the BP-RexMD and T-RexMD simulations. Also, very similar plots were obtained when using the T-RexMD trajectory for calculating the PCs or when plotting distribution projected onto PCs with smaller eigenvalues (not shown).

**Figure 4.**
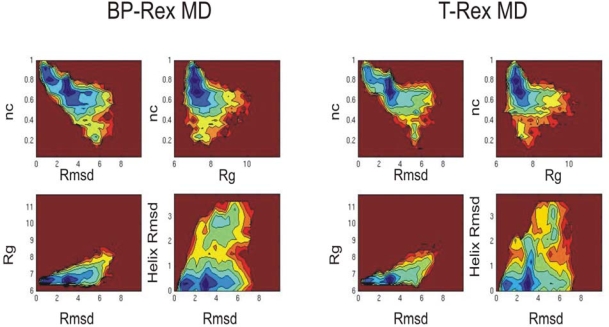
Free energy landscape for the Trp-cage conformations sampled in the reference replica during BP-RexMD and T-Rex MD simulations projected onto various combinations of the number of native contacts (nc), backbone Rmsd (Rmsd_Cα_), radius of gyration (Rg) and Rmsd_Cα_ (in Å) of the residues 2–8 from an α-helical structure. The logarithm of sampling density (low free energy) increases from red to blue.

In order to further characterize the folding free energy landscape of the Trp-cage protein it is also of interest to compare the probability distribution of sampled conformers (in the reference replica) for both types of RexMD as a function of reaction coordinates relevant for folding. Figure 4 shows 2D free energy contour maps (logarithm of the probability distributions in units of RT) using the sampled fraction of native contacts (nc), total Rmsd_Cα_, Rmsd_Cα_ of the α-helix and radius of gyration (Rg) as folding coordinates. Native contacts are defined as Cα-Cα atom pairs within 6.5 Å and the amino acids separated by more than 2 amino acids within the sequence. Again the results obtained by the BP-RexMD are very similar to the 2D plots derived from the T-RexMD indicating that both methods sample similar conformational states during the folding simulations.

As expected there is a clear correlation between total Rmsd_Cα_ and fraction of native contacts (Figure 4 top left corner in both cases). Interestingly, both simulations indicate a fraction of sampled conformers with approximately 80 % native contacts but an Rmsd_Cα_ of 3–4 Å from experiment separated from the sampled near native structures (with almost 100 % native contacts and an Rmsd_Cα_ of ~1 Å. There is also free energy barrier between the region near the native states and unfolded structures with Rmsd_Cα_ > 2 Å or fraction nc < 60%. The barrier is also observed in the other maps (e.g. nc vs. Rg or Rg vs. Rmsd_Cα_).

Interestingly, most of the sampled non-native structures have an Rg = 7–9 Å, only slightly larger than the native structure (Rg = 7 Å), but larger rmsd (> 4 Å) compared to the native structure. This clearly shows that the collapsed form of the structure occurs early in the simulation and agrees well with recent experiments by Mok *et al*. [41] which indicate a mostly collapsed unfolded state with average hydrodynamic radius of 8 Å (only slightly higher than the hydrodynamic radius of the native state ~7 Å [41]). The map for the Rmsd_Cα_ of the α-helix (residues 2–8) vs. total Rmsd_Cα_ clearly shows that structures were sampled with almost perfectly formed α-helix, low Rg but without formation of the complete native tertiary structure (total Rmsd_Cα_ < 2 Å). However, conformations without the α-helix but a total Rmsd_Cα_ not too far from the folded state were also populated in both simulations (Figure 4 bottom right corner).

**Figure 5.**
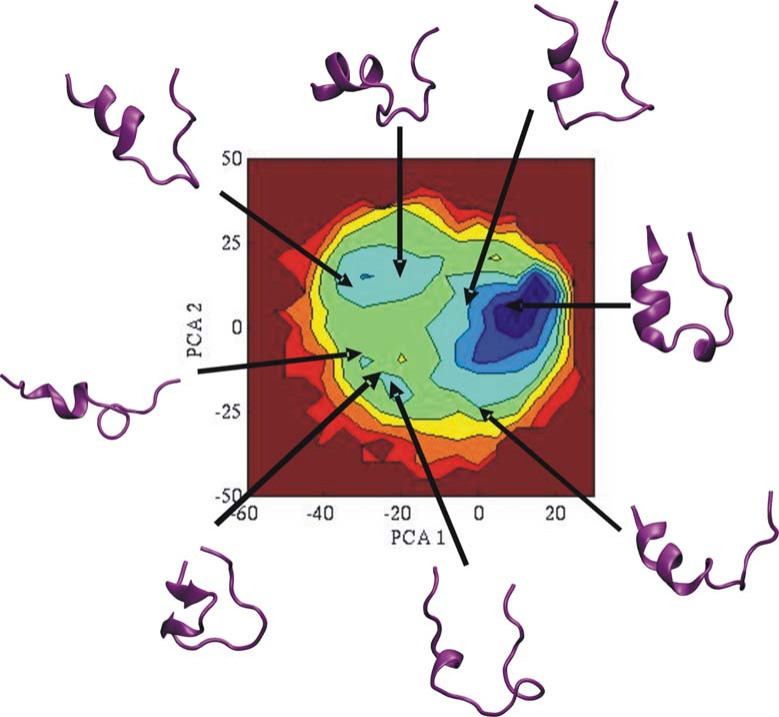
Mapping of representative Trp-cage conformations on populated regions in the 2D plane spanned by the two principal components with largest eigenvalue of the Cα positional covariance matrix (obtained from the BP-RexMD simulation). Trp-cage structures are shown as cartoon representation.

To further understand some of the interesting structural characteristics of Trp-cage folding, we looked at structures representing populated regions in the 2D plane spanned by the first two softest PCs (Figure 5). The most populated cluster (lowest free energy region colored blue in Figure 5) represents the native fold. Conformational changes associated with the softest PC correspond to an opening of the folded structure and partial disruption of the α-helix (a similar softest principal component was obtained by Paschek *et al*. in explicit solvent simulations [52,53]). The second PC represents a lateral screw type motion of the C-terminal part of the structure with respect to the α-helix. Interestingly, most of the significantly populated states are compact structures with some secondary structure elements already correctly formed (Figure 5).

### 3.3. Analysis of potential energy contributions to folding of Trp-cage protein

**Figure 6.**
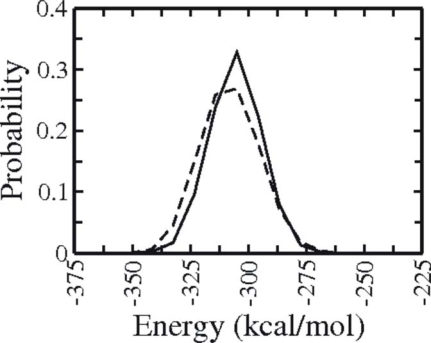
Distribution of the total potential energy of all sampled conformations from the BP-Rex MD simulation (continuous line) and T-Rex MD simulation (dashed line).

Although the projection of sampled states on PCs and various coordinates relevant for protein folding can give interesting hints on which physical and structural features are changing during structure formation the plots do not reveal information on the energetic driving forces for folding. To compare the sampling efficiency of BP-Rex MD and T-Rex MD simulation in terms of sampled potential energies the energy distribution was analyzed (Figure 6). Both simulations overall sample very similar total potential energies (distribution in Figure 6 includes all folded and unfolded structures sample during the entire simulations at the reference replica).

**Figure 7.**
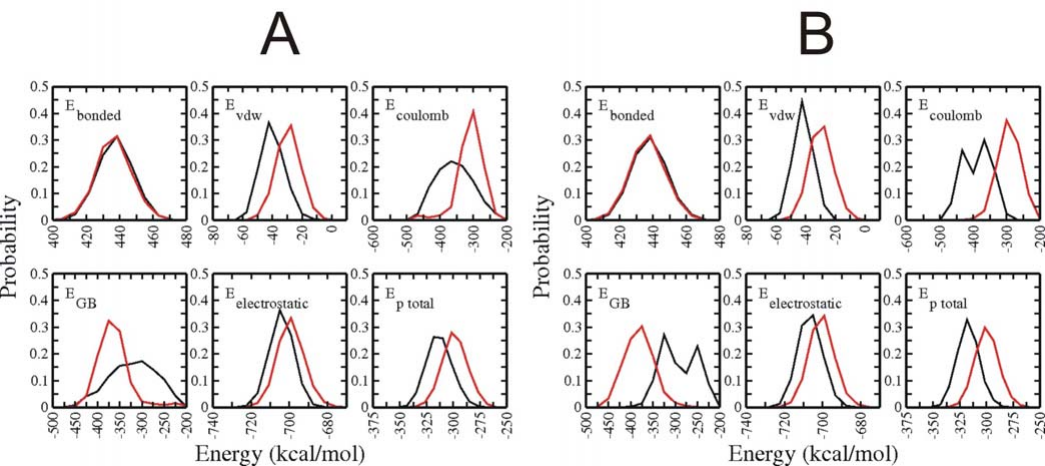
(A) Distribution of potential energy components to the force field comparing the first 5 ns (red line) and the last 5 ns (black line) of the BP-RexMD simulation (reference replica under control of original force field). E_bonded_ includes all energy contributions due to bond length, bond angles and dihedral angles in the protein. E_vdw_ represents the Lenard-Jones part of the force field energy. The E_electrostatic_ corresponds to the sum of Coulomb energy (E_Coulomb_) and electrostatic solvation based on a Generalized Born model (E_GB_). (B) same as in (A) except for conformations with a minimum Rmsd_Cα_ of 4.5 Å from the native structure representing unfolded structures (red line) or maximum Rmsd_Cα_ of 2.0 Å from the native structure representing folded structure (black line).

Besides of analyzing the total potential energy of the entire simulations it is of interest to look at force field energy contributions and comparing folded vs. unfolded Trp-cage conformations. This analysis is shown in Figure 7 for the BP-RexMD results (very similar plots were obtained for the T-RexMD, not shown). Two types of energy analysis were performed: In the first case (Figure 7a) the energy contributions of conformations sampled during the final 5 ns were compared with the energy contributions obtained for the first 5 ns simulation time (after a 0.5 ns equilibration run, see Methods). It is important to note, that even during the first 5 ns the simulations sampled to a large extend collapsed states with already significantly reduced surface area. However, only during the last 5 ns near native structures were sampled as dominant states (but not exclusively). In the second analysis (Figure 7b) only structures within 2 Å (RmsdCa) of the native state were considered as folded conformations (black line in Figure 7b) and were compared to unfolded states with an RmsdCa at least 4.5 Å from experiment. Both types of energy analysis give overall very similar trends and results. Interestingly, the distribution of the bonded energy contributions (bond length, bond angles and dihedral angles) are almost indistinguishable for both the folded and unfolded conformations. This indicates that folding does not involve any sterical strain of the sampled states. As expected the van der Waals energy (Lennard-Jones energy of the force field) distributions of the folded states (or structures sampled at the final stage of the simulation) is shifted to more negative (favorable) energies with respected to the unfolded states (Figure 7b) or structures sampled at the first 5 ns (Figure 7a). The van der Waals contribution favors folded states by 10–15 kcal mol^−1^ if one compares the maxima of the van der Waals distributions in Figures 7a or 7b. The van der Waals contribution is a measure of the sterical fit and overall compactness of the sampled structures. However, in the context of the present implicit solvent description it partially also represents hydrophobic contributions since van der Waals interactions with the solvent are not explicitly considered. The Coulomb part of the electrostatic energy strongly favors folding presumably due to hydrogen bond formation (α-helix and loop structure formation) and the salt bridge contact between residues Asp_9_ and Arg_16_. However, this contribution to the electrostatic energy is largely offset by an unfavorable electrostatic solvation upon going form folded to unfolded states. The electrostatic solvation is represented by the generalized Born (E_GB_) term. The calculations predict that overall the total electrostatic energy favors the folded states by an amount of ~6 kcal mol^−1^. One should keep, however, in mind that this contribution may depend on the exact definition of the boundary between protein and solution and on the parameters of the GB model. The energy analysis indicates that the folding transition shifts the total potential energy of the sampled states by ~18 kcal mol^−1^ (lower right panel in Figures 7a and 7b).

## 4. Conclusions

In order to systematically study structure formation processes of proteins it is necessary to overcome the limited conformational sampling on currently accessible time scales. Although the standard T-RexMD method is widely used to enhance conformational sampling it is limited to peptides or small proteins due to the rapid increase in the number of required replicas and increasing simulation time (to allow for sufficient exchanges among all replicas) with increasing system size. In the present study we have demonstrated that the recently developed BP-RexMD method [37] achieves very similar sampling quality as standard T-RexMD on the Trp-cage protein but at a fraction of the computational demand. It is expected that for larger systems especially including explicit solvent this advantage will be even more pronounced. An advantage of BP-RexMD compared to temperature RexMD is the fact the energy differences are only affected by the force field term that changes upon going from one replica to another replica run. Hence, the exchange probability is only affected by the backbone dihedral angle terms and not affected by solvent-solvent and solute-solvent (and many other solute-solute) contributions. The number of required replicas should only grow with the number of dihedral angles involved in application of the biasing potential. In both RexMD simulations conformations very close to the native state (Rmsd_Cα_ of the cluster centroid that represents the average structure of the most dominant conformational cluster sampled during the final part of the simulation in the reference replica was < 1.0 Å with respect to the native structure) were sampled after 20–30 ns. In contrast, in none of the five independent conventional MD simulations folding to near native structures was observed during 40 ns.

The simulation results on the mechanism of Trp-cage folding are in good qualitative agreement with available experimental results and with previous simulation studies. During the simulations an initial collapse at an early stage of the simulation was observed (during cMD as well as RexMD simulations). The analysis of the distribution of sampled conformers with respect to various possible folding coordinates revealed a conformational barrier (a region with reduced sampling) that separated the initially collapsed states from the fully folded native structure. Experimental studies using CINDP-NMR spectroscopy [41] suggested unfolded Trp-cage structures with hydrodynamic radius of ~8–9 Å similar to the range of Rg (7–10 Å) we found for the fraction of unfolded conformations. Experimental studies employing UV-resonance Raman spectroscopy [40] suggested the presence of α-helical structure even in the unfolded Trp-cage structures which agrees with the present simulation results of a significant fraction of helical segments even in the absence of a fully folded native structure.

The potential energy analysis of folded and unfolded protein structures indicated that both van der Waals and to a smaller extend electrostatic interactions favor folded Trp-cage conformations. As expected the change of the total potential energy upon Trp-cage folding by ~−18 kcal mol^−1^ is significantly larger than the free energy of folding which has been determined experimentally as ~−2.1 kcal mol^−1^ at 300 K [38]. Interestingly, the calculated change in potential energy corresponds quite well to the measured change in enthalpy upon folding of ~−13.5 kcal mol^−1^ based on differential scanning calorimetry [54]. The difference represents the conformational entropy that disfavors folding and needs to be outbalanced by the favorable change in internal energy of the Trp-cage protein. The study demonstrated that the BP-RexMD method allows for an efficient sampling of Trp-Cage conformations with fewer replicas compared to T-RexMD simulations and opens the possibility to use this method to study systematically the folding process of larger protein systems. However, it should be emphasized that the application to larger and more diverse proteins may require also further improvements in the force field and solvation model.
